# Treatment outcome and germline predictive factors of ropeginterferon alpha‐2b in myeloproliferative neoplasm patients

**DOI:** 10.1002/cam4.7166

**Published:** 2024-04-04

**Authors:** Chih‐Cheng Chen, Ming‐Chung Kuo, Ying‐Hsuan Wang, Sung‐Nan Pei, Ming‐Lih Huang, Chiu‐Chen Chen, Cih‐En Huang, Yi‐Yang Chen, Lee‐Yung Shih

**Affiliations:** ^1^ Division of Hematology and Oncology Chang Gung Memorial Hospital Chiayi Taiwan; ^2^ College of Medicine Chang Gung University Taoyuan Taiwan; ^3^ Division of Hematology‐Oncology Chang Gung Memorial Hospital at Linkou Taoyuan Taiwan; ^4^ Department of Hema‐Oncology E‐Da Cancer Hospital, I‐Shou University Kaohsiung Taiwan; ^5^ Division of Hematology and Oncology Da Chien General Hospital Miaoli Taiwan

**Keywords:** Asian, hematological responses, molecular responses, myeloproliferative neoplasm, polycythemia vera, ropeginterferon alpha‐2b, single nucleotide polymorphisms

## Abstract

**Background:**

Studies have shown that some single nucleotide polymorphisms (SNPs) could serve as excellent markers in foretelling the treatment outcome of interferon (IFN) in myeloproliferative neoplasms (MPN). However, most work originated from western countries, and data from different ethnic populations have been lacking.

**Methods:**

To gain insights, targeted sequencing was performed to detect myeloid‐associated mutations and SNPs in eight loci across three genes (*IFNL4*, *IFN‐γ*, and *inosine triphosphate pyrophosphatase* [*ITPA*]) to explore their predictive roles in our cohort of 21 ropeginterferon alpha‐2b (ROPEG)‐treated MPN patients, among whom real‐time quantitative PCR was also performed periodically to monitor the *JAK2*V617F allele burden in 19 *JAK2*V617F‐mutated cases.

**Results:**

ELN response criteria were adopted to designate patients as good responders if they achieved complete hematological responses (CHR) within 1 year (CHR1) or attained major molecular responses (MMR), which occurred in 70% and 45% of the patients, respectively. *IFNL4* and *IFN‐γ* gene SNPs were infrequent in our population and were thus excluded from further analysis. Two ITPA SNPs *rs6051702* A>C and *rs1127354* C>A were associated with an inferior CHR1 rate and MMR rate, respectively. The former seemed to be linked to grade 2 or worse hepatotoxicity as well, although the comparison was of borderline significance only (50%, vs. 6.7% in those with common haplotype, *p* = 0.053). Twelve patients harbored 19 additional somatic mutations in 12 genes, but the trajectory of these mutations varied considerably and was not predictive of any response.

**Conclusions:**

Overall, this study provided valuable information on the ethnics‐ and genetics‐based algorithm in the treatment of MPN.

## Graphical abstract

1. ROPEG induces exceedingly high rates of response in an Asia MPN cohort, including duration remission in cases who discontinued therapy.
2. Distinct genetic polymorphisms predicted responses in our patients, suggesting different ethnics‐based treatment algorithm needed.

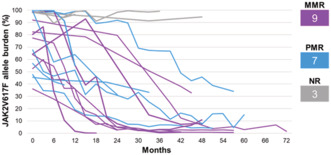

## 1 INTRODUCTION

For decades, interferon alpha (IFN‐α) has long been considered a valid treatment option in the management of classical *BCR*::*ABL1*‐negative myeloproliferative neoplasms (MPNs), which include essential thrombocythemia (ET), polycythemia vera (PV), and myelofibrosis (MF).^1^ Through inducing significant hematological, histopathological, and molecular responses, IFN‐α possesses potent disease‐modifying activities in MPN.^1^ However, considerable treatment‐related toxicity hinders its broader applicability. Recent advances in modified formulations (such as pegylated forms) drastically enhance the tolerability of IFN‐α and have thus expanded its use. The improved pharmacokinetic and toxicity profile is most notable in the novel, mono‐pegylated ropeginterferon‐alpha 2b (ROPEG), which owns exceptionally long half‐life and, resultantly, requires less frequent injections. As demonstrated in the phase 3 PROUD/CONTI‐PV study, it exhibits high tolerability and induces excellent clinical responses in PV patients.^2^ Considering the advantages of ROPEG and its superior efficacy, physicians have rekindled the enthusiasm in employing IFN‐α as a pivotal modality in the management of MPN.

On the other hand, despite the advance of IFN‐α therapy in MPN, some challenges still remain. For example, about one‐third of the patients did not respond well to ROPEG therapy in the PROUD/CONTI‐PV study.^2^ Given the financial, physiological, and psychological burdens associated with the treatment, identifying response predictors in treated patients has become an imperative clinical need. Lessons from hepatitis C, another important clinical indication in which IFN‐α therapy has been rather effective, have bought us to lights that several host‐ and treated‐related factors could portend the success of IFN therapy in these patients.^3^ Among all, genetic polymorphism is the most intriguing. Studies have shown that single nucleotide polymorphisms (SNPs) in several genes involved in IFN‐related signal pathways, such as IFN‐lambda (*IFN‐λ*,‐including *IFNL1*, *IFNL2*, *IFNL3*, and *IFNL4*) and IFN‐gamma (*IFN‐γ*), could be predictive for treatment response in patients with hepatitis C.^3^, ^4^ Interestingly, the polymorphic hereditary traits foretell not only the efficacy of IFN therapy but also the toxicity of the treatment, as SNPs in the inosine triphosphate pyrophosphatase (*ITPA*) gene induce more cytopenia in IFN‐treated hepatitis C patients.^5^, ^6^ Given that MPNs are diseases associated with excessive cytosis, it makes us wonder whether these *ITPA* SNPs could be response predictive markers in MPN patients instead of being markers of side effects.

Data have been comparably less while trying to employ genetic polymorphisms as guides for clinical decisions in MPN patients treated with IFN‐α. In a work done by Lindgren et al., the CC genotype of *rs12979860* in *IFNL3* gene was found to be associated with significantly superior complete hematological response (CHR) in PV patients treated with either IFN‐α 2a or 2b.^7^ More important information actually came from a subsidiary study of the PROUD/CONTI‐PV trial.^8^ Investigators from that study revealed that discrepant functional *IFNL4* diplotypes non‐redundantly exerted differential impacts on molecular response (MR) in those ROPEG‐treated patients when assessed with changes in *JAK2*V617F mutant allele burden (AB)^8^ These data pave ways for potential application of precision medicine in optimizing patient management during IFN therapy for MPN.

However, one caveat to apply the aforementioned information into our clinical practice lies in the ethnic discrepancies.^3^ It is uncertain whether the response prediction effects of specific SNPs could be extrapolated into different ethnic groups. Moreover, the frequency of certain genetic polymorphisms varies drastically across divergent racial populations,^9^ which makes some response predictors useless in populations with low variability in those specific genetic loci. Importantly, with most of the studies originated from Western countries, data from Asia–Pacific region are lacking. Thanks to a compassionate use program provided by the manufacturer of ROPEG (PharmaEssentia, Taiwan), we have a cohort of MPN patients who have been receiving continuous ROPEG therapy for years. To provide contrasting perspectives on appraising the impacts of genetic variations on the clinical outcome of ROPEG‐treated MPN patients, next generation sequencing (NGS) was applied to explore further. We also probed into the collaborating non‐driver mutations in these patients to see their effects on therapeutic responses as well as their trajectories along the course of ROPEG therapy. In the end, we were able to identify unique genetic variants that successfully predicted the efficacy and adverse effects of ROPEG therapy in our MPN patients.

## 2 MATERIALS AND METHODS

### 2.1 Patients and diagnosis

In this study, 21 ROPEG‐treated MPN patients were included from two branches (Linkou and Chiayi) of Chang‐Gung Memorial Hospital, Taiwan. The diagnosis of MPN and subtype stratification was based on the 2016 World Health Organization classification of myeloid neoplasms.^10^ At diagnosis, driver mutations in *CALR* exon 9 and *MPL* genes were assessed by polymerase chain reaction (PCR) amplification followed by bidirectional sequencing. Real‐time allele‐specific quantitative PCR assay was used to detect *JAK2*V617F mutation and monitor the changes in AB throughout their clinical courses. The study was approved by the Institutional Review Board (IRB) of our hospital (IRB approval number: 202201220B0C602).

### 2.2 Treatment and response assessment

For the treatment, ROPEG was given every 2 weeks at a starting dose of 250 μg. If there were no significant side effects, the dose of this agent would be increased by 100–150 μg every 2 weeks until it reached the target dose of 500 μg on Week 4 or 6. Hemograms, biochemistry profiles, and adverse events (AEs) were routinely monitored and recorded at every visit for each patient. *JAK2*V617F AB in the peripheral blood granulocytes were quantified periodically. All AEs were graded according to the National Cancer Institute's Common Terminology Criteria for Adverse Events version 5.0 (CTCAE v5.0). Relevant clinical data before and after treatment were collected for comparison and analysis.

**FIGURE 1:**
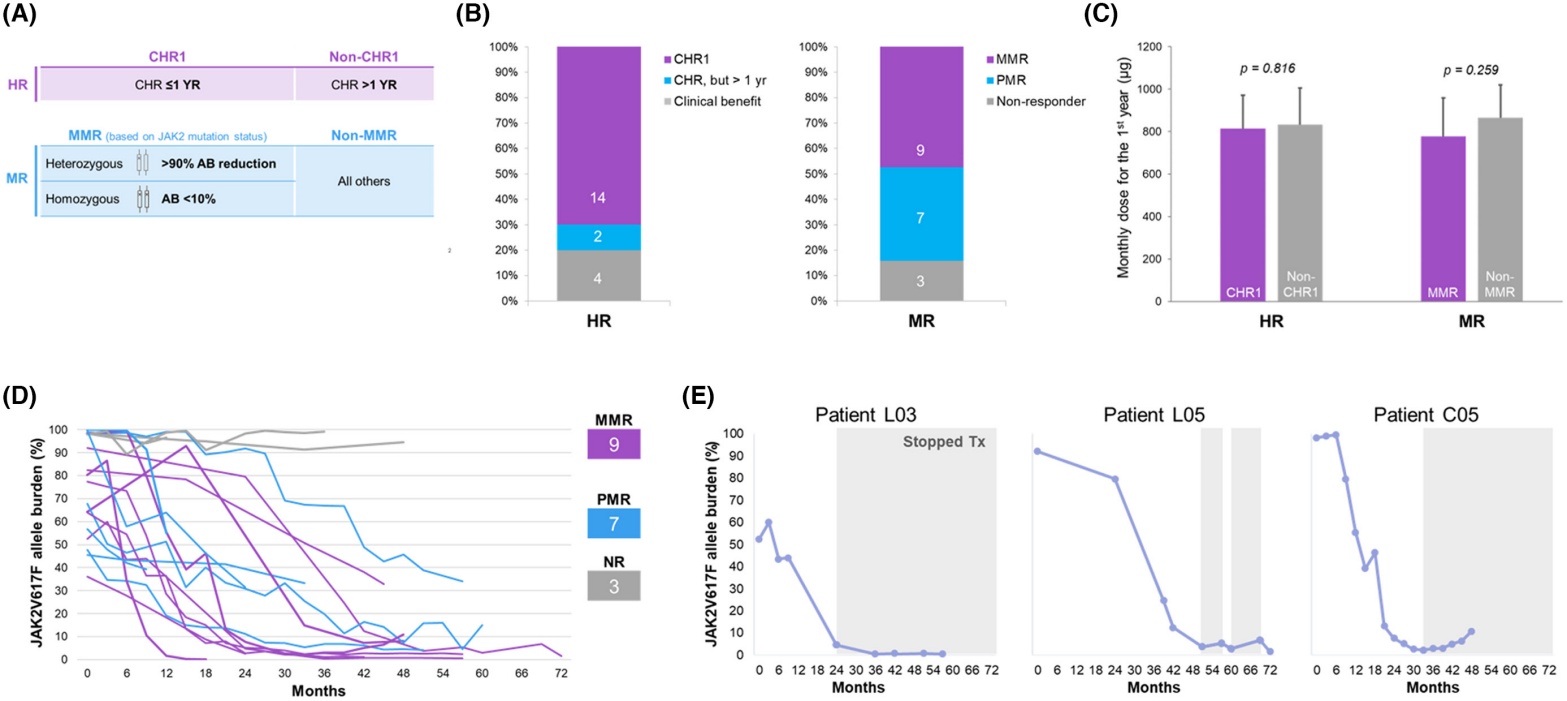
Response and clinical outcomes of ROPEG‐treated MPN patients. (A) CHR1: achievement of complete hematological response (CHR) within 1 year. MMR: major molecular response. Homozygous *JAK2*V617F allele burden (AB) below 10% post‐treatment and heterozygous mutation attaining more than 90% AB reduction as MMR responders. (B) Number of cases with hematological response (HR) and molecular response (MR). (C) Association between ROPEG doses and responses. The average monthly dose for the first year was compared between those with achieving either CHR1 or MMR and poor responders. (D) Patterns of MR in 19 *JAK2*‐mutated cases. *JAK2*V617F AB are plotted with time on ROPEG treatment. Patients with MMR, partial MR (PMR), and no response were represented in dark violet, aqua, and light gray colors, respectively. (E) The dynamic changes of AB in 3 *JAK2*‐mutated patients who discontinued ROPEG therapy for various reasons. The shaded area represents the time when the treatment was stopped or interrupted.

Owing to high CHR and MR rates in our patient cohort, we decided to re‐classify them with highly stringent criteria. Therefore, for response assessment, ELN response criteria for CHR^11^ was adopted to stratify patients into good responders (CHR1, if CHR was achieved within 1 year) or poor responders (non‐CHR1). The achievement of MR was defined as having post‐treatment mutant AB decreased by more than 20% as compared to the baseline levels. We categorized cases with homozygous *JAK2*V617F mutation achieving post‐treatment AB below 10% and those with heterozygous mutation attaining more than 90% reduction as major MR (MMR) responders, the remainders as non‐MMR responders (Figure 1A). Achievement of MMR represented a highly desirable outcome, whereas non‐MMR responders included two populations of patients—those who did not respond at all (non‐responders), and those with a MR but not reaching the criteria of MMR (categorized as partial MR responders, Figure 1B).

### 2.3 Targeted sequencing

Targeted sequencing by NGS on a Miseq instrument was performed to detect myeloid‐associated mutations and SNPs in eight loci across three genes (*IFNL4*, *IFN‐γ*, and *ITPA*; the SNPs are listed in Table S1). The mutational profile was determined using SOPHiA Myeloid Solution™ panel (v2, 51 genes) and subsequently analyzed with SOPHiA DDM platform software in pre‐ and post‐treatment samples.

### 2.4 Statistical analysis

The two‐tailed independent Student's *t*‐test was used to compare continuous variables between two groups. For comparison of dichotomous variables, a Pearson chi‐squared or a Fisher's exact test (for expected values of >5 or ≤5, respectively) was applied. All calculations were performed using the Statistical Package of Social Sciences software (SPSS, Inc., Chicago, IL, USA) and GraphPad Prism 7.0 (GraphPad Software, Inc., San Diego, CA, USA). The level of statistical significance was set at 0.05 for all tests.

## 3 RESULTS

### 3.1 Baseline characteristics of the patients

Table 1 lists the detailed clinical information of the 21 enrolled patients. At baseline, the median age of these patients was 45.6 years (range: 15.7–86.4 years), and the median disease duration was 5.0 years (range: 0.5–25.5 years). Among the patients, there were one case of ET, 15 cases of PV, 2 cases of pre‐fibrotic PMF, and three cases of post‐PV/ET MF. Of the five prePMF and post PV‐ET MF patients, four were treated for poorly controlled myeloproliferation as demonstrated on their hemogram, whereas the fifth case had intermediate (int)‐1‐risk secondary MF (SMF) (based on MYSEC‐PM model^12^) and elected to receive ROPEG therapy for its disease‐modifying potential in spite of showing no signs of excessive cytosis. The one ET case, a 37‐year‐old male, received ROPEG treatment because of extreme thrombocytosis (>1500 × 10^9^/L) that was poorly controlled by anagrelide. For the 15 PV patients, the reason of ROPEG therapy included high‐risk diseases (five patients, including four with either hydroxyurea‐resistant or–intolerant diseases), poor tolerance to phlebotomy (five cases), and patients' preference (five cases). *JAK2*V617F was the driver mutation in 19 cases, and the remaining two patients had *CALR* (exon 9)‐mutated prePMF and triple negative ET, respectively. The median baseline *JAK2*V617F AB was 80.2% (range: 36.1%–99.9%). The average monthly ROPEG doses in those patients were 841 and 624 μg in the first year and from the second year on, respectively (data were not shown).

**TABLE 1:** Clinical information of enrolled MPN patients.

Case	Age (years)	Gender	Disease duration (years)	Thrombosis history	Diagnosis	Driver mutation	Mutant allele burden^a^
L01	78.0	Male	25.5	Yes	Post‐PV MF	*JAK2*V617F	98.3
L02	56.7	Female	11	No	Post‐PV MF	*JAK2*V617F	98.4
L03	42.8	Male	9.9	No	PV	*JAK2*V617F	52.5
L04	15.7	Female	7.9	No	PV	*JAK2*V617F	45.5
L05	28.1	Female	1.6	No	PV	*JAK2*V617F	92.1
L06	34.9	Female	8.5	No	Pre‐PMF	*CALR* Exon 9	26.9^b^
L07	86.4	Female	19.9	Yes	PV	*JAK2*V617F	82.4
L08	70.6	Female	18.5	Yes	PV	*JAK2*V617F	64.3
C01	78.3	Male	1.3	No	PV	*JAK2*V617F	67.7
C02	69.8	Male	4.3	Yes	PV	*JAK2*V617F	47.7
C03	49.8	Male	8	No	PV	*JAK2*V617F	98.9
C04	37.4	Female	3	No	PV	*JAK2*V617F	77.4
C05	38.3	Female	4	No	PV	*JAK2*V617F	98.3
C06	42.7	Female	0.7	No	PV	*JAK2*V617F	63.9
C07	45.6	Male	0.8	No	PV	*JAK2*V617F	80.2
C08	67.1	Male	2.6	No	PV	*JAK2*V617F	99.9
C09	36.4	Male	13	No	ET	Triple negative	–
C10	66.6	Male	5	No	Pre‐PMF	*JAK2*V617F	98.0
C11	30.2	Female	4	No	Post‐ET MF	*JAK2*V617F	56.7
C12	49.2	Male	16	No	PV	*JAK2*V617F	99.4
C13	44.6	Female	0.5	No	PV	*JAK2*V617F	36.1

*Note*: ^a^ All data were obtained by real‐time quantitative allele‐specific PCR assay, except for the one with *CALR* mutation,^b^ in which the VAF was calculated based on the results of NGS.

### 3.2 Treatment efficacy and toxicities

Excluding the SMF case without excessive cytosis, 20 ROPEG‐treated patients were evaluable for hematological response. CHR was achieved within 1 year (CHR1) in 14 (70%) patients, whereas two patients achieved CHR after 1 year (Figure 1B, left panel), which made an overall CHR rate of 80%. Although the remaining four patients did not achieve CHR, clinical benefit was observed. The median time to CHR was 5 months (range: 1–45 months). Among patients with *JAK2* mutation, nine cases (47.4%) achieved MMR and seven additional patients had partial MR, which made an overall MR rate of 84.2% in the 19 patients (Figure 1B, right panel). The median time to MMR was 24 months (range: 12–51 months). The average monthly dose within the first year of therapy was tested as a factor in predicting treatment outcomes. However, there was no apparent association between the dose and efficacy (Figure 1C), both in the regards of HR and MR.

Among these patients, the treated‐related AEs were generally mild (Table 2). Most of them were either grade 1 or 2 toxicity, with fatigue and alopecia being more common. Grade 3/4 pancytopenia occurred in the int‐1‐risk SMF patient who had less adequate marrow reserve and was treated for disease modification purpose. She achieved partial MR 8 months after ROPEG treatment and then went on to receive allo‐transplant at her own discretion. The remaining prominent AE was grade 3 transaminitis in a patient who had severe fatty liver and impaired liver function at baseline. The side effect was easily manageable, although dose reduction was required in this case. All but one patient suffered from thrombotic events after initiation of ROPEG therapy. This was an 86‐year‐old high‐risk PV patient with concurrent comorbidities including diabetes and hypertension who had already suffered from stroke twice prior to ROPEG treatment. After initiation of therapy, she had another stroke episode at Week 32 and a transient ischemic attack (TIA) event (fully recovered within 24 h) at Week 113. Considering the facts that the patient did not achieve CHR until Week 42 and her MR had not been documented until 34 weeks into ROPEG therapy, we believed the stroke episode at Week 32 could be partially attributable to her active PV disease. Furthermore, old age and underlying cardiovascular risk factors (diabetes and hypertension) could constantly put the patient at risk of recurrent TIA or stroke attacks even if her PV was under well control.

**TABLE 2:** Toxicities related to treatment.

	Gr 1–2	Gr 3–4
Hematological		
Neutropenia	2 (9.5%)	1 (4.8%)
Anemia	1 (4.8%)	1 (4.8%)
Thrombocytopenia	1 (4.8%)	1 (4.8%)
Non‐hematological		
Transaminitis	4 (19%)	1 (4.8%)
Hypothyroidism	2 (9.5%)	0
Fatigue	4 (19%)	0
Alopecia	4 (19%)	0
Musculoskeletal	3 (14.3%)	0
Depression	1 (4.8%)	0
Insomnia	1 (4.8%)	0
Diarrhea	1 (4.8%)	0

To appraise the pace of MR, the dynamic changes in *JAK2*V617F AB across the observed period are demonstrated in Figure 1D. Three non‐responders had persistently high *JAK2*V617F AB, whereas all the remaining patients showed an encouraging trend of reduction in the mutant clone. Specifically, the decline in *JAK2* mutant AB was particularly steep in those who achieved MMR (Figure 1D, purple lines). Among those who achieved MMR, three patients discontinued ROPEG therapy for various reasons in spite of the deep MR. Figure 1E illustrates the changes in *JAK2*V617F AB with time in these patients. The shaded area represents the time when the treatment was stopped or interrupted. The first patient remained in continuous MMR after nearly 3 years of discontinuing therapy (left panel). The second patient, treated irregularly during the late course because of a drug supply issue, remained similarly stable in spite of treatment interruption (middle panel). Notably, the MR could be further improved once the therapy was re‐initiated in this patient, a phenomenon observed twice during the course of treatment. The mutant clone seemed to expand gradually in the third case (right panel), who discontinued ROPEG therapy because of the achievement of MMR and the desire for pregnancy. However, the clonal expansion was probably clinically irrelevant, as the patient remained in CHR status and the mutant AB stayed below 10% at the time of this writing.

### 3.3 Genetic polymorphisms as response predictors

**FIGURE 2:**
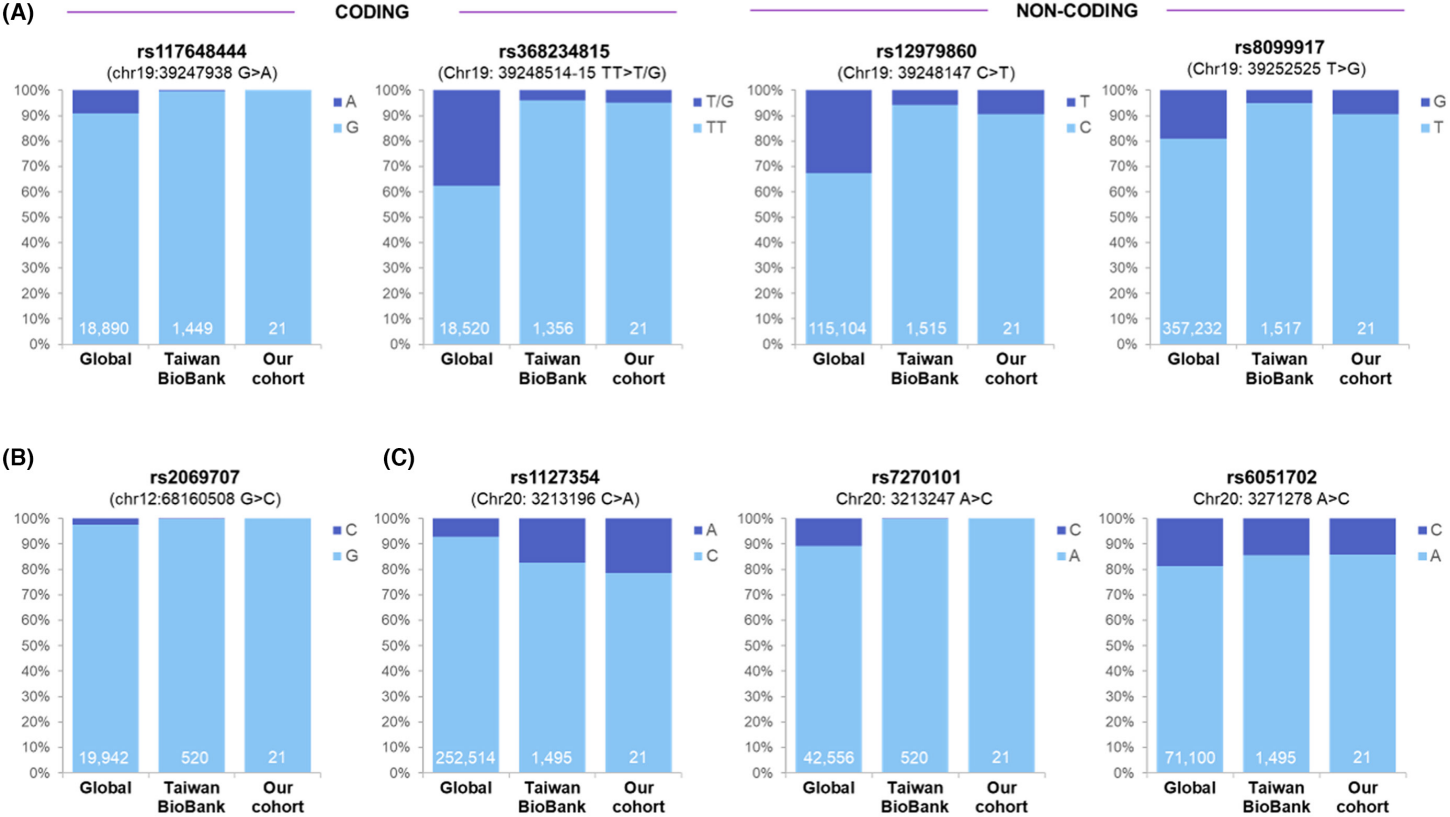
Prevalence of genetic polymorphisms in genes of interests across different populations. (A) Polymorphisms in the *IFNL4* gene. Four SNPs, including two in the coding region (*rs117648444* G>A and *rs368234815* TT>T/G) and two in the non‐coding region (*rs12979860* C>T and *rs8099917* T>G). Data from global database, Taiwan Biobank, and our cohort are shown. The number at the bottom of each column indicated the population size of each cohort. (B) Genetic polymorphism in the *IFN*‐*γ*‐gene (*rs2069707* G > C). (C) Polymorphisms in the three loci of *ITPA*‐*γ*‐gene, including one in the coding region (*rs1127354* C>A) and two intron variants (*rs7270101* A>C and *rs6051702* A>C).

**FIGURE 3:**
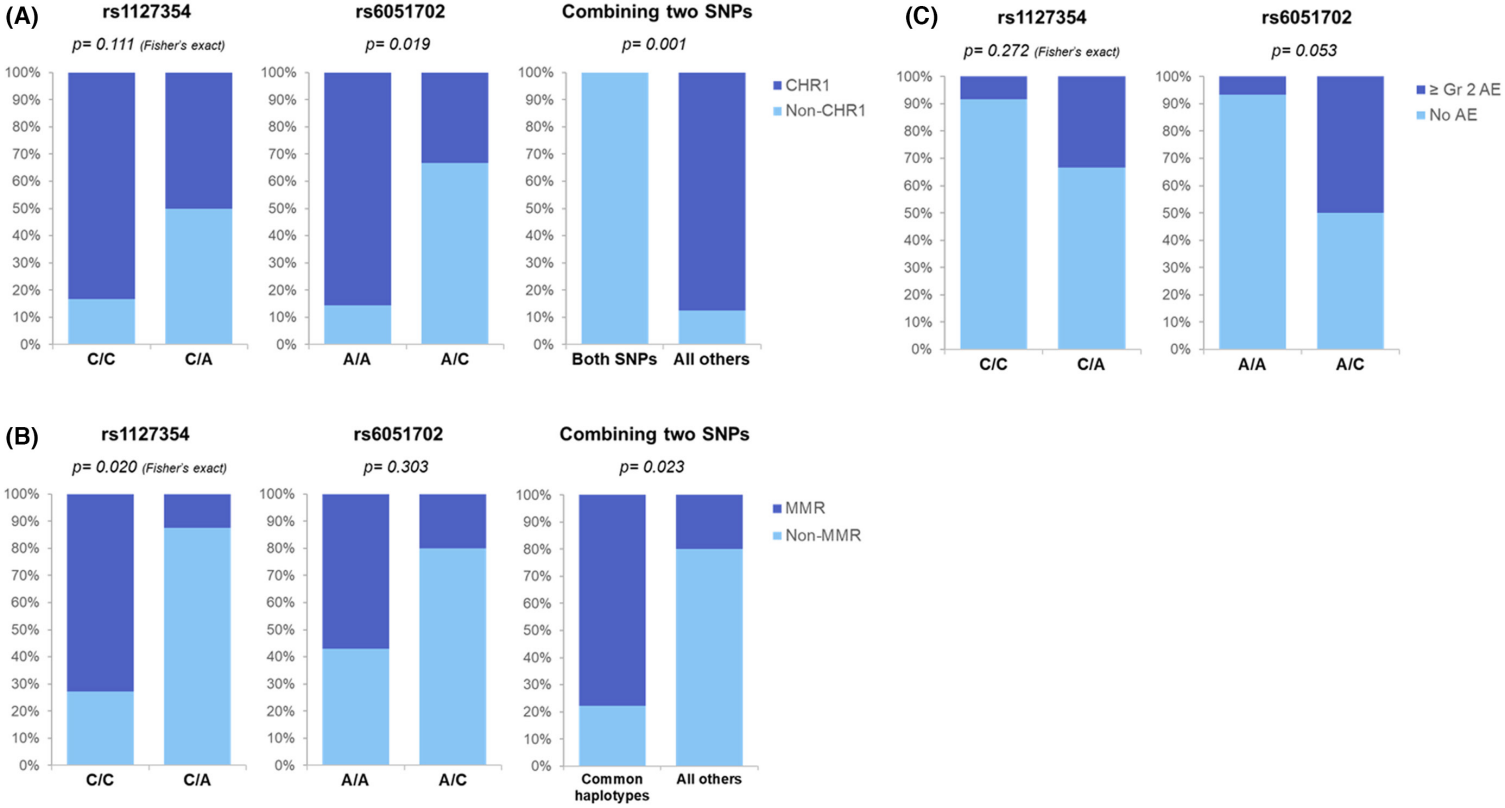
The roles of *ITPA* polymorphisms in predicting the efficacy of ROPEG therapy in MPN patients. (A) Hematological response **(**HR) prediction with *ITPA* SNPs. CHR1: achieving complete HR within 1 year. (B) Molecular response (MR) prediction with *ITPA* SNPs. MMR: major MR. (C) Hepatotoxicity prediction with *ITPA* SNPs. Grading was based on CTCAE v5.0.

Four *IFNL4* SNPs were assessed and two public databases including NCBI dbSNP (Build 156 released on September 21 2022) and Taiwan Biobank (https://taiwanview.twbiobank.org.tw/; assessed on August 10 2023) were used for comparison. As illustrated in Figure 2A, the frequencies of polymorphisms in *rs117648444* G>A and *rs368234815* TT>T/G in the coding region (left panel), and *rs12979860* C>T and *rs8099917* T>G in the non‐coding region (right panel), were lower both in our cohort and in the Taiwan Biobank when compared to those in the global database. As a result, the *IFNL4* SNPs could not be used as response predictors because of the limited case number harboring those genetic variants. Similarly, the prevalence of *IFN‐γ rs2069707* G>C polymorphism was too low in our ethnic population to perform further predictive analysis (Figure 2B). On the other hand, among the three *ITPA* polymorphisms studied, two were fairly common in our patient cohort (Figure 2C); *rs1127354* locates within the coding region and *rs6051702* A>C is an intron variant. These two SNPs were used to assess their association with response. As demonstrated in Figure 3A, *rs6051702* A>C polymorphism was correlated with an inferior CHR1 rate (middle panel), whereas *rs1127354* C>A did not play a significant role in response prediction (left panel). In the meantime, on combining these two SNPs, it was observed that they together portended a dismal clinical outcome, as all the individuals who carried both polymorphisms failed to achieve CHR1 (right panel). On analyzing the relationship between SNPs and MR, only 19 *JAK2*‐mutated patients were included, constituting the major composition of the cohort. The results showed that *rs1127354* C>A was associated with an inferior MMR rate (Figure 3B, left panel, *p* = 0.020), yet the comparison on the impact of MMR achievement between the *rs6051702* common haplotype and its variant was not statistically significant (Figure 3B, middle panel). On combining these two SNPs together, patients who did not harbor variants in both loci were more likely to be MMR responders than their counterparts (77.8% vs. 20%, Figure 3B, right panel, *p* = 0.023).

On exploring the association between genetic polymorphisms and treatment‐related adverse events, it was observed that individuals who carried *rs6051702* A>C polymorphism had a trend to develop ROPEG‐associated grade 2 or higher hepatotoxicity (Figure 3C) when compared to those with the common haplotype (50% vs. 6.7%, *p* = 0.053). Among the four (including three female) patients who experienced grade 1 alopecia, no apparent association between the occurrence of this adverse effect and genetic polymorphisms could be identified (data were not shown).

### 3.4 The trajectory of co‐existing mutations throughout the treatment course and their impacts on treatment outcomes

**FIGURE 4:**
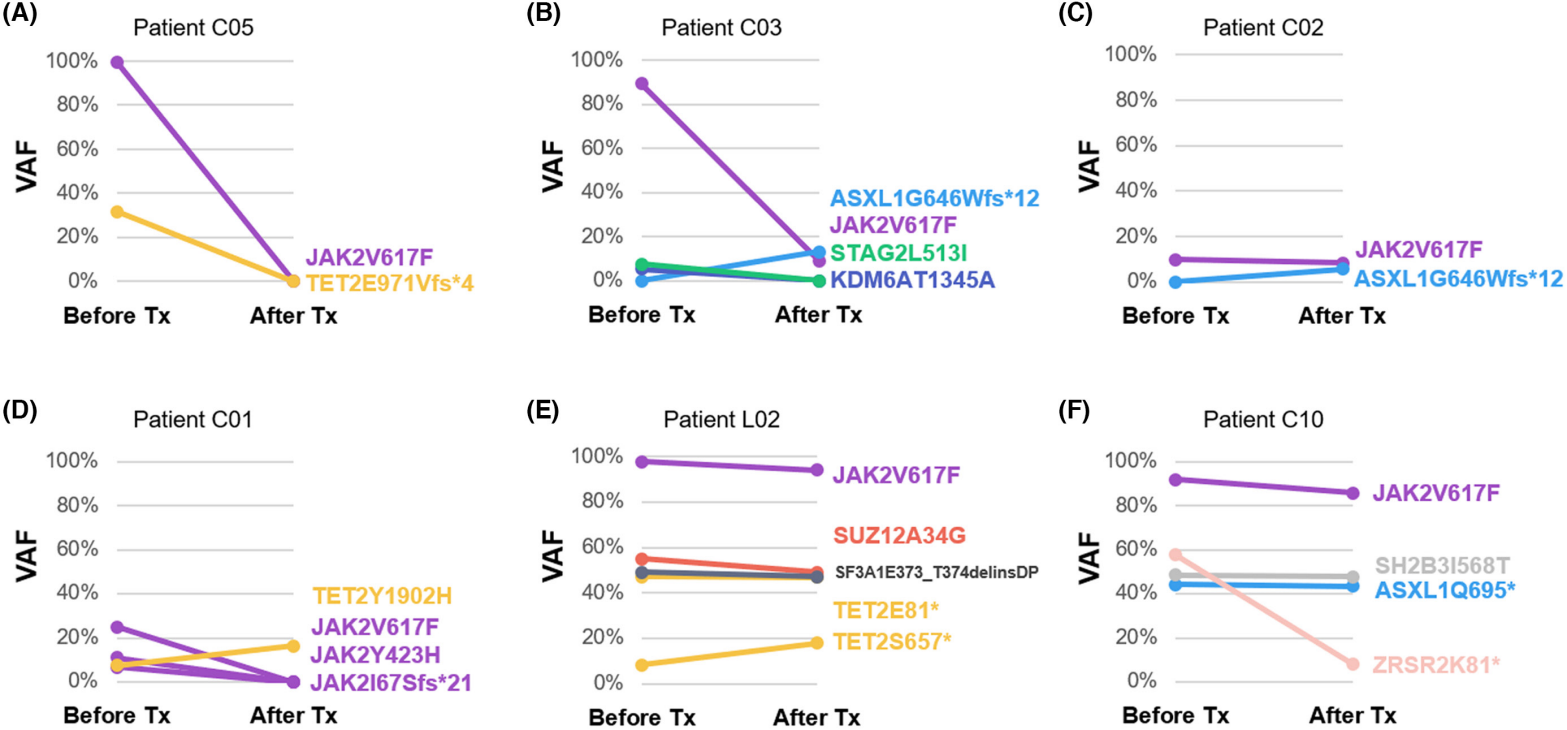
Clonal changes of gene mutations through the treatment courses in representative cases. (A–F) Baseline and post‐treatment changes in variant allele frequencies (VAF) in co‐existing mutations in six selective cases.

On targeted sequencing analysis, 12 patients were identified harboring 19 additional somatic mutations in 12 genes (*ASXL1*, *TET2*, *SH2B3*, *ZRSR2*, *KDM6A*, *SMC3*, *NF1*, *SUZ12*, *JAK3*, *SF3A1*, *STAG2*, and *BCOR*, Table S2). The presence of these mutations was not associated with the rates of either CHR1 or MMR (data were not shown). These co‐existing mutations remained mostly stable throughout the treatment course, with some notable exceptions (Figure 4). Complete eradication of co‐existing mutants was achieved in three clones after ROPEG therapy: *TET2*E971Vfs*4 mutation in a patient with MMR on the *JAK2* clone (Figure 4A), and both *KDM6A*T1345A and *STAG2*L513I in a second patient whose MR on *JAK2*V617F was graded as partial (Figure 4B). Conversely, clonal evolution was detected in the second case (Figure 4B) and another patient (Figure 4C) who developed a new *ASXL1* mutation (G646Wfs*12) at 39 and 48 months after the initiation of ROPEG therapy, respectively. One patient had complete MR in three different *JAK2* mutations, but a small *TET2*Y1902H clone slightly expanded in this case (Figure 4D). In two cases of non‐molecular responders, both harbored several mutant clones that responded poorly to ROPEG therapy (including the *JAK2* clone) (Figure 4E,F).

## 4 DISCUSSION

Approved in the United States and Europe, ROPEG has emerged as a promising therapy for patients with PV. Although a recent study has adequately addressed the issue on the health‐economics perspective of this expensive agent,^13^ we believe that there are merits to identify those who are more likely to respond well to the drug to make the treatment more cost‐effective and to avoid the unnecessary suffering among those who might not benefit from the therapy. This actually constitutes the motivation of our current study. By understanding which factors would predict a superior response, physicians could tailor their treatment plans accordingly. Additionally, through incorporating NGS on key myeloid genes, this study provides valuable information that would help unravel the complex interplay among the treatment, the disease, and the driver as well as the collaborating mutations.

Although the *IFNL4* diplotype was proved to be the decisive factor in predicting ROPEG‐induced MR in the PROUD‐PV cohort,^8^ the results of that study could not be applied in our patients, largely because of the discrepant prevalence of several key polymorphisms across various ethnic cohorts (Figure 2). Generally, genetic variations can be highly specific to particular ethnic groups, reflecting unique evolutionary paths, environmental influences, and historical migrations. These disparities in genetic makeup may lead to divergent expressions of genes and varied responses to treatment. Alternatively, the variants may function differently across distinct ethnic populations. Although the information on MPN is scarce, relevant reports on the contrasting association between genomics and IFN‐related treatment response in HCV patients are abundant. For example, different variants in the human leukocyte antigen (HLA) genes have been found to predict contrary viral responses in IFN‐treated HCV patients among various populations.^14^, ^15^ Similarly, polymorphisms in the vitamin D receptor gene may affect IFN‐associated HCV response differently in Western and Asian populations.^16^, ^17^, ^18^ These differences highlight the intricate nature of genetic polymorphisms in predicting the success of IFN therapy in human diseases. Importantly, these facts also illustrate the critical need for region‐specific research in pharmacogenomics, recognizing that one‐size‐fits‐all approaches may not be appropriate when dealing with diverse ethnic backgrounds.

In our ROPEG‐treated cohort, this study reported a high CHR rate (80%) and MR rate (84%). It should be mentioned that only 19 *JAK2*‐mutated patients were included for MR assessment. We expected that such a strategy would make the identified response predictive markers more reliable and clinically applicable, as the involved patients were more homogenous, at least on the molecular aspect with regards to the disease biology. Compared to the 20%–70% CHR rates and 30%–60% MR rates across several prospective clinical trials using various formulations of IFN‐α in MPN during the last two decades (Table 3), these responses were significantly better than those seen in most of the studies. It is inappropriate to compare the results across different trials, especially considering the retrospective nature of this study and the heterogeneous populations of patients involved. However, the unusually high response rates bring to our attention on the exploration of potential reasons. Among all, ethnic differences in the frequencies of genetic polymorphism might play significant roles in this aspect, and variants in the *IFNL4* gene could be key factors. The *IFNL4 rs12979860* CC genotype has been identified as a predictor for a better MR rate in the PROUD/CONTI‐PV study.^8^ Considering the genotype is more prevalent in Taiwanese population than in Western countries (Figure 2A), it is not surprising to witness a better response in our patients. Similar phenomenon could be seen in the experience of IFN therapy in hepatitis C patients, as the CC genotype was found to be more prevalent in Asian populations^9^ that contributed to the higher response rates in these ethnic cohorts.^4^ Second, the different formula and doses of IFN‐α used for MPN patients could lead to disparate outcome as well. As demonstrated in Table 3, patients treated with the traditional, short‐acting IFN‐α had the lowest CHR and MR rates, whereas those receiving the ultra‐long acting ROPEG enjoyed a more encouraging benefit. The once weekly, long‐acting pegylated IFN‐α (PEG‐IFNα)‐treated MPN patients had a response rate somewhere in between (Table 3). The estimated average monthly doses of IFN‐α were estimated at around 180–360 μg and 500–1000 μg for PEG‐IFNα‐ and ROPEG‐treated patients, respectively, which possibly resulted in seemingly better response in these latter cases. This could also be reflected in our patient cohort, as the high average monthly ROPEG doses (841 and 624 μg in the first year and from the second year on, respectively) potentially contributed to our excellent CHR and MR rates.

**TABLE 3:** Published prospective clinical trials on IFN‐α in MPN.

	Trial (ref)	Type of IFN	Patients^a^	Dosing	HR	MR
Short‐acting	Huang et al.^19^	IFNα‐2b	64 PV 123 ET	300 MU×3/week	CHR PV: 29.7% (19/64) ET: 28.5% (35/123)	PV 54.7% (35/64) No data on ET
Long‐acting	Kiladjian et al.^20^	PEG‐IFNα‐2a	40 PV	90–180 μg/week	CHR 94.6% (35/37) at 1 year	93.1% (27/29) at 2 years
	Masarova et al.^21^	PEG‐IFNα‐2a	43 PV 40 ET	90–450 μg/week (mostly 90–270)	CHR at 7 years: PV: 77% ET: 73%	MR at 7 years: 63% for PV 37% for ET
	DALIAH^22^, ^23^	PEG‐IFNα‐2a PEG‐IFNα‐2b	82 each for two types of IFN‐α	2a: 45 μg/week; 2b: 35 μg/week	CHR 36% at 1.5 years	28%–29% at 3 years
	MPD‐RC 111^24^	PEG‐IFNα‐2a	50 PV 65 ET	45–180 μg/week	CR at 1 year^a^ 22% for PV 43% for ET	41.3%
	MPD‐RC 112^25^	PEG‐IFNα‐2a	43 PV 39 ET	45–180 μg/week	CR at 1 year^a^ 28% for PV 44% for ET	NA
Ultra‐long acting	PROUD‐PV^2^ CONTI‐PV	ROPEG	127 PV 95 PV	50,100,150,200…500 μg/q2week	CHR 54.6% at 6 years	66% at 6 years
	Low‐PV^26^, ^27^	ROPEG	64 PV	100 μg/q2week	Hct Control: 83% (43/52) at 2 years	55% (16/29) at 2 years
	Edahiro et al.^28^	ROPEG	29 PV	50,100,150,200…500 μg/q2week	CHR: 52% (15/29) at 1 year	42% (11/26) at 1 year
	Lee et al.^29^	ROPEG	99 PV	250–350–500 μg/q2week	CHR: 81% at Week 60	70% at Week 60
	Current study^b^	ROPEG	21 MPN	250–350–450‐500 μg/q2week	CHR 80%	MR 84%

Abbreviations: ET, essential thrombocythemia; HR, hematological response; MPN, myeloproliferative neoplasms; MR, molecular response; NA, not available; PV, polycythemia vera.

^a^ CR definition based on ELN criteria.^11^

^b^ Retrospective.

Aside from the driver mutations of *JAK2*, *CALR*, and *MPL*, MPNs are also characterized by the presence of concomitant somatic mutations in a handful of genes.^30^ Importantly, some of these genetic alterations provide prognostic information in either assessing the risk of disease progression or foreboding the potential phenotypic manifestations. However, the role of these co‐existing mutations in predicting response to cytoreductive therapy remains undefined, and their dynamic changes under IFN‐based therapy in MPN is an evolving area of research. Data from the DALIAH trial,^22^ a phase three randomized controlled trial of IFN‐α (including IFNα‐2a and IFNα‐2b) versus hydroxyurea (HU) in MPN patients, brought to light the most important information in this regards up to date. Genomic profiling by NGS was performed in paired pre‐ and post‐treatment samples (IFN‐α, *n* = 101; HU, *n* = 34) in that study.^31^ Although treatment‐emergent *DNMT3A* mutations were observed more commonly in patients treated with IFN‐α (especially in those not attaining CHR), the investigators could not identify any role that non‐driver mutations may play in predicting therapy response or resistance in IFN‐treated patients.^31^ This study, enrolled less patients who were nevertheless uniformly treated, had similar findings in demonstrating that these coexisting mutations were not correlated with either HR or MR. Diverse clonal patterns were also found as there were regression/loss, expansion, or acquisition of mutant clones in our ROPEG‐treated patients. Additionally, the response patterns in those co‐occurrent mutations correlated poorly with those seen in the driver mutations. The results from DALIAH trial and our cohort suggest that the variations of these non‐driver mutations, as well as their interactions with driver genes, were highly complex and heterogeneous. Further study to fully understand their implications in the context of IFN‐α treatment in larger cohorts of MPN patients is warranted.

From our experience, the use of ROPEG was associated with low toxicities, and grade 1 or 2 transaminitis was among the most commonly observed (19%) side effects in our cohort. The incidence of impaired liver function was similar to that seen in the PROUD/CONTI‐PV study.^2^ The association between liver toxicity and ROPEG could be complex and multifaceted. The monopegylated form of ROPEG results in altered metabolism of IFN‐α and a higher in vivo concentration with prolonged exposure of the drug in the liver, potentially leading to liver toxicity.^32^ Alternatively, liver injury could be caused by immune reactions, as autoimmune manifestations were common side effects associated with IFN therapy.^33^ In addition, genetic factors might as well influence a person's susceptibility to such an injury, which prompted us to explore into the association between genetic polymorphisms and ROPEG‐associated hepatotoxicity, among all other side effects. This study revealed that individuals harboring *ITPA rs6051702* A>C polymorphism could be more likely to suffer from treatment‐related elevated levels of transaminases. However, hampered by the limited number of overall patients and the low incidence of this toxicity associated with ROPEG, the comparison was not statistically significant (*p* = 0.053). It would be intriguing to see if future study could attest or refute such a correlation.

In our cohort, we had three patients who discontinued ROPEG therapy for various reasons. They all had deep MR at the time of cessation and, after a median follow‐up of 24 months into the “treatment‐free” period, all of them remained in CHR and had the *JAK2* mutant AB below 10%. Whether IFN‐α therapy should be discontinued in those who respond well and, if discontinuation is deemed appropriate, when the treatment could be stopped are still topics of debate, especially considering that the experience on ROPEG is still very limited worldwide. In a retrospective study reporting the long‐term outcome of 293 MPN patients who discontinued IFN therapy, two factors were associated with persistent CHR: a CHR duration of more than 2 years and a *JAK2*V617F VAF below 10% at the time of discontinuation.^34^ In spite of the limited case number, these three patients in our cohort nevertheless provided further supportive evidence showing that treatment cessation might be feasible in those who achieve excellent MRs. However, further collaborative studies on the long‐term outcomes, potential risks, and overall survival of MPN patients with post‐interferon cessation are needed to balance between the risks and benefits of continuous treatment.

The clinical applicability of this study is admittedly restrained in several aspects. The retrospective nature of this work may be susceptible to biases due to incomplete collection of relevant information, which could hinder the ability to control for confounding variables. Further, the limited case number of our MPN patients could make the analytical outcome ambiguous, and the lack of a validation cohort also diminishes the reliability of our findings. Additionally, although PV constituted the major disease subtype (71.4% of the patients) in our cohort, the patient composition was still slightly heterogeneous. We did have that in mind but just intended to provide different ethnic perspectives from what we learned from the PROUD‐PV study.

In summary, this study provides valuable information on the ethnics‐and genetics‐based approach in the treatment of MPN. The results on using genetic polymorphisms (specifically *ITPA* SNPs) to predict efficacy and adverse events in our MPN patients treated with ROPEG show promise in improving personalized treatment. Through NGS analysis on sequential samples, this study is also able to demonstrate the clonal patterns of co‐existing mutations and their lack of impacts on clinical outcomes. Shall the data be validated in additional studies, it may allow for optimizing patient management in the context of IFN‐α therapy for MPN patients.

## AUTHOR CONTRIBUTIONS

**Chih‐Cheng Chen:** Conceptualization (equal); data curation (equal); funding acquisition (equal); project administration (equal); resources (equal); writing – original draft (equal); writing – review and editing (equal). **Ming‐Chung Kuo:** Data curation (equal); formal analysis (equal); investigation (equal). **Ying‐Hsuan Wang:** Formal analysis (equal); investigation (equal); visualization (equal). **Sung‐Nan Pei:** Data curation (equal); investigation (equal). **Ming‐Lih Huang:** Data curation (equal); investigation (equal). **Chiu‐Chen Chen:** Data curation (equal); investigation (equal). **Cih‐En Huang:** Data curation (equal); investigation (equal). **Yi‐Yang Chen:** Data curation (equal); investigation (equal). **Lee‐Yung Shih:** Conceptualization (equal); funding acquisition (equal); resources (equal); supervision (equal); writing – original draft (equal); writing – review and editing (equal).

## FUNDING INFORMATION

The study was supported by grants from the Chang‐Gung Memorial Hospital to C‐C Chen (CORPG6K0013 and COPRG6K0023) and to L‐Y Shih **(**DOH‐102‐TD‐C‐111‐006, NSTC112‐2314‐B‐182‐055, and XPRPG3H0011–16**).**

## CONFLICT OF INTEREST STATEMENT

The authors have no conflict of interest.

## ETHICS STATEMENT

Approval of the research protocol by an Institutional Reviewer Board: This work was approved by the Institutional Review Board of Chang‐Gung Memorial Hospital (IRB approval number: 202201220B0C602) in accordance with the Declaration of Helsinki.

## INFORMED CONSENT

All participants provided informed consent.

## Supporting information


Tables S1–S2.

## Data Availability

All relevant data are available from the corresponding authors upon reasonable request.
