# Supplementary material for: Treatment outcome and germline predictive factors of ropeginterferon alpha‐2b in myeloproliferative neoplasm patients
Source: Cancer Med. 2024 Apr 4;13(7):e7166. doi: 10.1002/cam4.7166 (PMC10993704; doi:10.1002/cam4.7166)
Supplement: Supplementary file 1 — Tables S1–S2. [file CAM4-13-e7166-s001.docx]

**Supplementary Table 1:**

**Supplementary Table 1. Information on SNPs studied in the current work.**

| SNP | Gene | Position (GRCh38.p14) | Alleles | Consequence |
| --- | --- | --- | --- | --- |
| rs117648444 | *IFNL4* | chr19:39247938 | G>A | Missense Variant |
| rs368234815 | *IFNL4* | chr19:39248514-39248515 | TT>T/G | Frameshift Variant |
| rs12979860 | *IFNL4* | chr19:39248147 | C>T | Intron Variant |
| rs8099917 | *IFNL4* | chr19:39252525 | T>G | None |
| rs2069707 | *IFN-γ* | chr12:68160508 | G>C | 2KB Upstream Variant |
| rs1127354 | *ITPA* | chr20:3213196 | C>A | Missense Variant |
| rs7270101 | *ITPA* | chr20:3213247 | A>C | Intron Variant |
| rs6051702 | *ITPA* | chr20:3271278 | A>C | Intron Variant |

The Reference SNP (rs) Report is available from the public database, NCBI dbSNP Build 156. Chr, chromosome.

**Supplementary Table 2:**

**Supplementary Table 2. Co-existing variants prior to treatment with ROPEG in the patient cohort**

| Case | Diagnosis | Driver Mutation | Co-existing variants at baseline |
| --- | --- | --- | --- |
| L01 | Post-PV MF | *JAK2*V617F | *SH2B3*S337*, *MPL*M602T, *JAK3*R222H |
| L02 | Post-PV MF | *JAK2*V617F | *TET2*E81*, *TET2*S657*, *SUZ12*A34G, *SF3A1*E373_T374delinsDP |
| L03 | PV | *JAK2*V617F | - |
| L05 | PV | *JAK2*V617F | - |
| L04 | PV | *JAK2*V617F | - |
| L07 | PV | *JAK2*V617F | *BCOR*D1331Y |
| L08 | PV | *JAK2*V617F | *SMC3*S1074N |
| L06 | Pre-PMF | *CALR* Exon 9 | *ZRSR2*R421Q |
| C01 | PV | *JAK2*V617F | *JAK2*I67Sfs*21, *JAK2*Y423H, *TET2*Y1902H |
| C02 | PV | *JAK2*V617F | *ASXL1*646Wfs*12 |
| C03 | PV | *JAK2*V617F | *KDM6A*T1345A, *STAG2*L513I, *ASXL1*646Wfs*12 |
| C04 | PV | *JAK2*V617F | - |
| C05 | PV | *JAK2*V617F | *TET2*E971Vfs*4 |
| C06 | PV | *JAK2*V617F | *KDM6A*E860K |
| C07 | PV | *JAK2*V617F | *JAK2*A331G |
| C08 | PV | *JAK2*V617F | - |
| C09 | ET | Triple negative | *NF1*P678S |
| C10 | Pre-PMF | *JAK2*V617F | *ASXL1*Q695*, *SH2B3*I568T, *ZRSR2*K81* |
| C11 | Post-ET MF | *JAK2*V617F | - |
| C12 | PV | *JAK2*V617F | - |
| C13 | PV | *JAK2*V617F | - |
